# Urinary DNA methylation‐based risk stratification model to triage patients for repeat transurethral resection of bladder tumours

**DOI:** 10.1002/ctm2.1549

**Published:** 2024-01-22

**Authors:** Wei Ouyang, Ran Xu, Hanyu Yao, Shusuan Jiang, Qiang Lu, Cai Lv, Pei Li, Genming Xu, Jianye Liu, Long Wang

**Affiliations:** ^1^ Department of Urology The Third Xiangya Hospital Central South University Changsha China; ^2^ Department of Urology The Second Xiangya Hospital Central South University Changsha China; ^3^ Department of Urology Hunan Cancer Hospital The Affiliated Cancer Hospital of Xiangya School of Medical Central South University Changsha China; ^4^ Department of Urology Hunan Provincial People's Hospital First Affiliated Hospital of Hunan Normal University Changsha China; ^5^ Department of Urology Affiliated Haikou Hospital of Xiangya Medical College Central South University Haikou China; ^6^ Yearth Biotechnology Co. Ltd. Changsha China

Dear Editor,

Bladder cancer (BCa) is one of the most common malignancies of the urinary tract, with over 570 000 new cases diagnosed worldwide every year. Approximately 80% of BCa is non‐muscle invasive BCa (NMIBC). Transurethral resection of bladder tumours (TURBT) is the first‐line approach for diagnosing and treating NMIBC. Some studies have demonstrated that the tumour residual rate after initial TURBT varies from 4% to 78%. Moreover, the positive rate for repeat TURBT (re‐TURBT) varies widely and is highly correlated with the effectiveness of the initial surgery.^1^ These data suggest that some patients might undergo unnecessary re‐TURBT. Therefore, there is an urgent need to develop a noninvasive tool to detect residual tumours, thereby avoiding unnecessary operations and reducing associated costs. Recently, various panels based on urine biopsy have been employed to detect BCa. Our previous study showed that the methylation status of *NRN1* exhibits remarkable performance in detecting urothelial carcinoma and can be used for its diagnosis.^2^, ^3^ In this prospective, multicenter study, we developed a novel risk‐stratification model based on urine biopsy to identify residual tumours after initial TURBT and to avoid unnecessary re‐TURBT procedures.

To develop a urine‐based, noninvasive assay, we analyzed 124 patient samples from two independent cohorts: a training cohort of 78 patients from five centres and a validation cohort of 46 patients from another centre (ChiCTR2100043328). The methods of sample collection, DNA extraction and DNA methylation detection are described in our previously published study.^3^, ^4^ The clinical characteristics of enrolled cases are shown in Table S1. The residual rate after initial TURBT was 30.77% (24/78). The urinary *NRN1* methylation demonstrated remarkable detection efficacy (Figure 1A). The receiver operating characteristic (ROC) curve showed an area under curve (AUC) of .87, 83.33% sensitivity, 90.74% specificity, 80.00% positive predictive value (PPV) and 92.54% negative predictive value (NPV), respectively.

**FIGURE 1:**
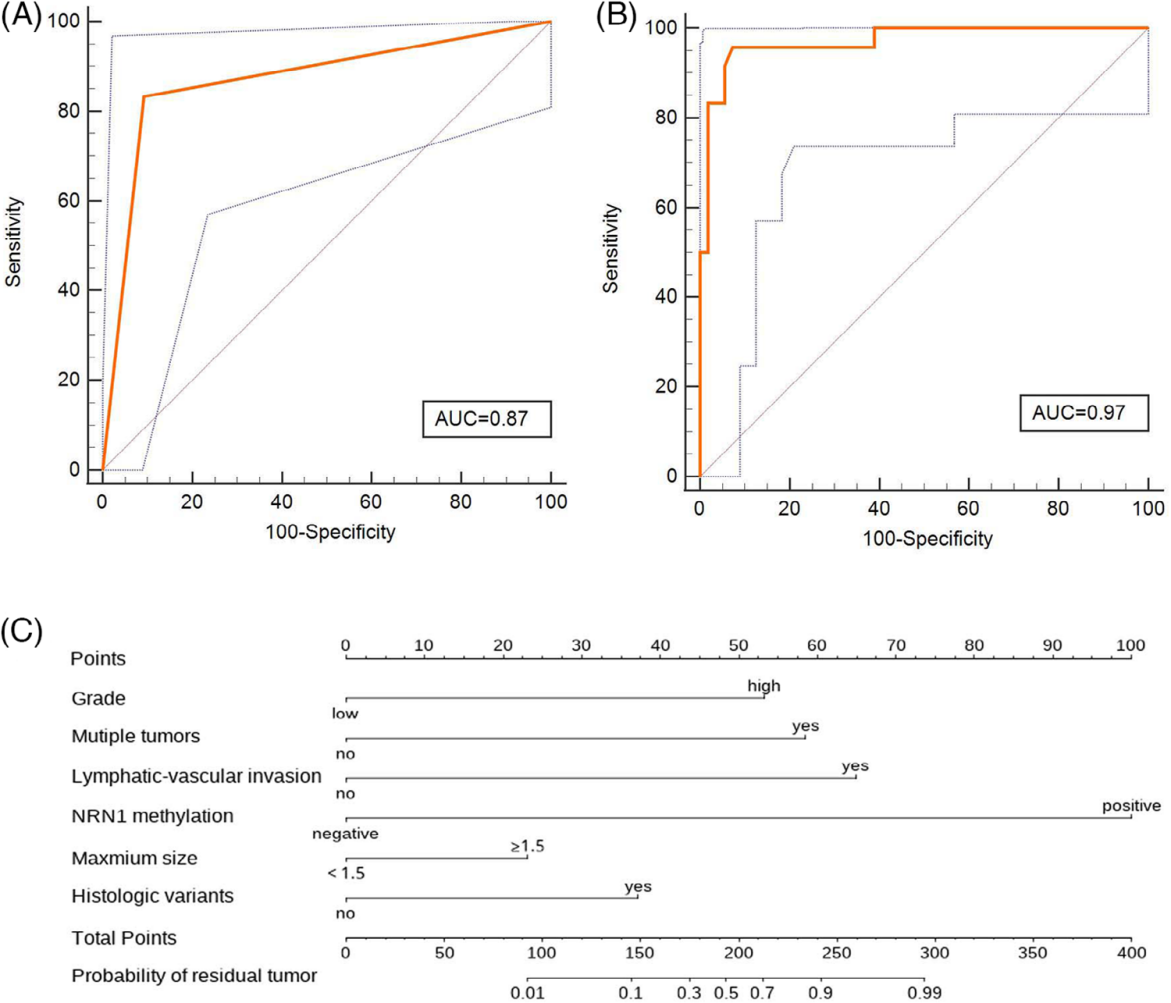
Training phase of the risk assessment model for the identification of residual disease after initial TURBT. (A) ROC curve for *NRN1* methylation from training cohort patients. The AUC, sensitivity and specificity are .87, 83.33% and 90.74%, respectively. (B) ROC curve for the risk assessment model from training cohort patients. The AUC, sensitivity and specificity are .97, 95.83% and 92.59%, respectively. (C) A nomogram illustrating the probability of residual disease risk. For clinical purposes, the scores of each covariate are added, and the total score is depicted on the total score point axis. AUC, area under the curve; ROC, receiver operating characteristic; TURBT, transurethral resection of bladder tumours.

Logistic regression analysis was used to evaluate the correlation of clinical characteristics (lymphatic‐vascular invasion, tumour grade, maximum tumour size, histologic variants and multiple tumours) with the residual tumour (Table S2). Lymphatic‐vascular invasion, multiple tumours and urinary *NRN1* methylation were significantly influential in detecting residual tumours. To further improve the efficiency of residual tumour detection, we built a risk assessment model incorporating urinary *NRN1* methylation, tumour grade, the presence of multiple tumours, maximum tumour size, histologic variants and lymphatic‐vascular invasion (Figure 1C). Interestingly, our risk assessment model demonstrated remarkable efficiency in detecting residual tumours (odds ratio: 1384.11; *p* < .01). Therefore, we conducted the ROC curve analysis for this model (Figure 1B). This model achieved a favourable AUC of .97 (*p* < .01), outperforming the AUC of .87 for the urinary *NRN1* methylation alone. Moreover, this model showed optimal accuracy, with 95.83% sensitivity, 92.59% specificity, 85.20% PPV and 98.06% NPV. This model showed that the largest AUC at the estimated risk for residual tumour exceeds 25%. The reliability of the risk assessment model was tested using an external validation cohort of 46 patients. In this cohort as well, the model showed excellent performance (Figure 2), achieving .96 AUC, 93.75% sensitivity, 93.33% specificity, 88.27% PPV and 96.66% NPV.

**FIGURE 2:**
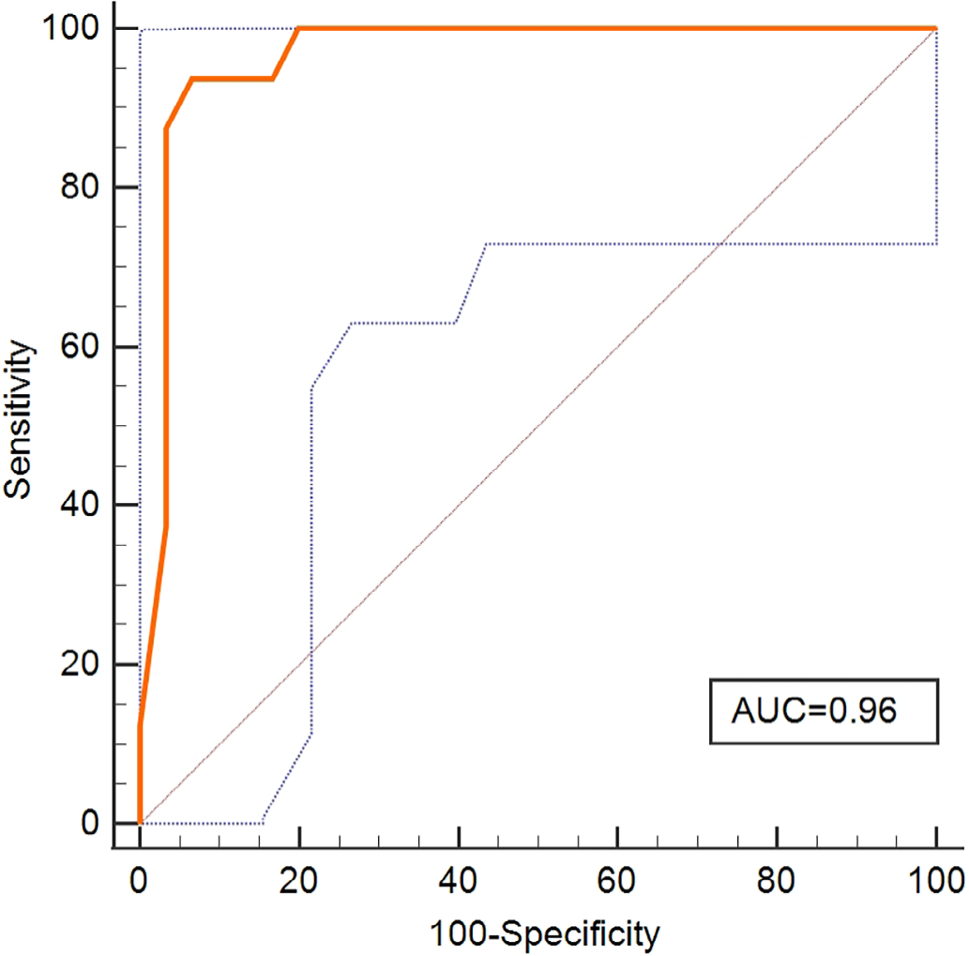
Validation phase of the risk assessment model for the identification of residual disease after initial TURBT. The risk assessment model showed excellent performance, with an AUC of .96, a sensitivity of 93.75%, a specificity of 93.33%, a PPV of 88.27% and an NPV of 96.66%. AUC, area under the curve; NPV, negative predictive value; PPV, positive predictive value; TURBT, transurethral resection of bladder tumours.

According to the literature, the main mechanisms of NMIBC recurrence are the presence of residual disease after TURBT and tumour reimplantation.^5^ The presence of residual tumours after TURBT can increase the risk of disease progression and cancer‐specific mortality.^6^ Therefore, the ability to detect such residual tumours is crucial to predict disease recurrence and to identify patients who require re‐TURBT.

As compared to cystoscopy, urine sediment DNA is a noninvasive tool, and shows promising efficacy in the detection of BCa.^7^ Some diagnostic tools, such as UcSeek,^8^ UroSEEK^9^ and utMeMa,^10^ have been used to detect early‐stage BCa, and they have shown high accuracy. Furthermore, utMeMa has also shown a capacity to detect residual disease, achieving 86.7% accuracy, 90.0% sensitivity and 83.1% specificity. We assessed the urinary *NRN1* methylation status by methylation‐specific polymerase chain reaction, a cost‐effective method, and our risk‐stratification model yielded favourable results in residual tumour detection, with a sensitivity of 95.83%. We subsequently developed an optimal risk assessment model combining urinary *NRN1* methylation with these clinical features. The model showed excellent diagnostic value for detecting residual tumours (AUC = .97), at a cut‐off of 25%. Approximately two‐thirds of patients (29 of 46; 63.04%) could potentially avoid re‐TURBT, and only one man (3.45%) in the validation cohort might have a missed residual tumour. Our results showed that the urinary *NRN1* methylation‐based risk stratification model has tremendous clinical potential for detecting residual tumours, thereby drastically reducing the number of unnecessary re‐TURBT procedures. By accurately identifying true high‐risk patients and sparing others from unnecessary re‐TURBT, both patient distress and the associated costs can be reduced.

In this study, we devised a novel noninvasive risk assessment model that exhibited high accuracy in detecting residual tumours. Although all enrolled patients underwent re‐TURBT, only 32.36% (40 of 124 [24 in the training set and 16 in the validation set]) of patients exhibited positive postsurgical pathology, indicating that approximately two‐thirds of patients underwent unnecessary re‐TURBT procedures. According to this newly established risk assessment model, only 3.23% of patients are overtreated, which dramatically reduced the number of surgeries.

In conclusion, this risk stratification model is a potential tool to triage patients with NMIBC for re‐TURBT, thereby reducing the distress of unnecessary surgery and the associated costs. Importantly, this is the first study to integrate various important clinical variables into a nomogram designed to detect residual disease after TURBT, providing valuable assistance to urologists during the decision‐making process.

## AUTHOR CONTRIBUTIONS

Wei Ouyang had full access to all the data in the study and took responsibility for the integrity of the data and the accuracy of the data analysis. *Study concept, design, funding acquisition, administrative or material support and study supervision*: Long Wang. *Acquisition, analysis and interpretation of data*: Shusuan Jiang, Hanyu Yao, Qiang Lu and Cai Lv. *Drafting of the manuscript and statistical analysis*: Wei Ouyang. Critical revision of the manuscript for important intellectual content: all authors. *Technical support*: Pei Li and Genming Xu. All authors have read and agreed to the published version of the manuscript.

## CONFLICT OF INTEREST STATEMENT

The authors declare no conflicts of interest.

## ETHICS STATEMENT

This study was conducted in accordance with the Declaration of Helsinki and approved by The Ethical Committee of Third Xiangya Hospital of Central South University (Changsha, China) (No. 2020‐S336, 11 June 2020).

## CONSENT FOR PARTICIPATION AND PUBLICATION

Informed consent was obtained from all subjects involved in the study before participation as well as for the publication of this article.

## Supporting information

Supporting Information

## Data Availability

The data used to support the findings of this study are available from the corresponding author upon request.

## References

[1] Zurkirchen MA , Sulser T , Gaspert A , Hauri D . Second transurethral resection of superficial transitional cell carcinoma of the bladder: a must even for experienced urologists. Urol Int. 2004;72:99‐102. 14963348 10.1159/000075961

[2] Ouyang W , Luo L , Zhang J , et al. Urine cellular DNA point mutation and methylation for identifying upper tract urinary carcinoma. Cancers (Basel). 2022;14 :3537. 35884598 10.3390/cancers14143537 PMC9319988

[3] Zhang J , Xu R , Lu Q , Xu Z , Liu J , Li P , et al. A novel methylation marker NRN1 plus TERT and FGFR3 mutation using urine sediment enables the detection of urothelial bladder carcinoma. Cancers (Basel). 2023; 15 :615. 36765573 10.3390/cancers15030615 PMC9913436

[4] Ou Z , Li K , Yang T , et al. Detection of bladder cancer using urinary cell‐free DNA and cellular DNA. Clin Transl Med. 2020;9:4. 31938901 10.1186/s40169-020-0257-2 PMC6960275

[5] Bryan RT , Collins SI , Daykin MC , Zeegers MP , Cheng KK , Wallace DM , et al. Mechanisms of recurrence of Ta/T1 bladder cancer. Ann R Coll Surg Engl. 2010;92:519‐524. 20522307 10.1308/003588410X12664192076935 PMC3182798

[6] Bishr M , Lattouf JB , Latour M , Saad F . Tumour stage on re‐staging transurethral resection predicts recurrence and progression‐free survival of patients with high‐risk non‐muscle invasive bladder cancer. Can Urol Assoc J. 2014;8:E306‐310. 24940455 10.5489/cuaj.1514 PMC4039592

[7] Murtaza M , Dawson SJ , Tsui DW , et al. Non‐invasive analysis of acquired resistance to cancer therapy by sequencing of plasma DNA. Nature. 2013;497:108‐112. 23563269 10.1038/nature12065

[8] Wang P , Shi Y , Zhang J , et al. UCseek: ultrasensitive early detection and recurrence monitoring of urothelial carcinoma by shallow‐depth genome‐wide bisulfite sequencing of urinary sediment DNA. EBioMedicine. 2023;89:104437. 36758479 10.1016/j.ebiom.2023.104437 PMC9941055

[9] Springer SU , Chen CH , Rodriguez Pena MDC , et al. Non‐invasive detection of urothelial cancer through the analysis of driver gene mutations and aneuploidy. Elife. 2018;7:e32143. 29557778 10.7554/eLife.32143 PMC5860864

[10] Chen X , Zhang J , Ruan W , et al. Urine DNA methylation assay enables early detection and recurrence monitoring for bladder cancer. J Clin Invest. 2020;130:6278‐6289. 32817589 10.1172/JCI139597 PMC7685755

