# Supplementary material for: Urinary DNA methylation‐based risk stratification model to triage patients for repeat transurethral resection of bladder tumours
Source: Clin Transl Med. 2024 Jan 22;14(1):e1549. doi: 10.1002/ctm2.1549 (PMC10802133; doi:10.1002/ctm2.1549)
Supplement: Supplementary file 1 — Supporting Information [file CTM2-14-e1549-s001.docx]

**Table S1. Clinical characteristics of clinical cohorts**

| **Characteristic** | **Training cohort** | **Validation cohort** | ***P* value** |
| --- | --- | --- | --- |
| **Age, y**  Mean (SD) | 62.43(9.09) | 63.85(9.62) | 0.42 |
| **Gender, n (%)**  Male  Female | 64 (82.05)  14 (17.95) | 38 (82.61)  8 (17.39) | 0.94 |
| **Methylation, n (%)**  Positive  Negative | 25 (32.05)  53 (67.95) | 16 (34.78)  30 (65.22) | 0.76 |
| **Grade, n (%)**  Low grade  High grade | 30 (38.46)  48 (61.54) | 14 (30.43)  32 (69.57) | 0.37 |
| **Multiple tumor**  Yes  No | 42 (53.85)  36 (46.15) | 25 (54.35)  21 (45.65) | 0.96 |
| **Lymphatic-vascular invasion**  Yes  No | 47 (60.26)  31 (39.74) | 27 (58.70)  19 (41.30) | 0.86 |
| **Maximum tumor size (cm)**  ＜1.5  ≥1.5 | 42 (53.85)  36 (46.15) | 20 (43.48)  26 (56.52) | 0.27 |
| **Stage**  Ta and Tis  T1 | 3 (3.85)  75 (96.15) | 1 (2.17)  45 (97.83) | 1.00 |
| **Histologic variants**  no  yes | 66 (84.62)  12 (15.38) | 41 (89.13)  5 (10.87) | 0.48 |

SD: standard deviation;

**Table S2.** Univariate and multivariate logistic regression analysis for residual tumor

| Variables | Univariate analysis | | |  | Multivariate analysis | | |
| --- | --- | --- | --- | --- | --- | --- | --- |
|  | OR | 95%CI | *P* |  | OR | 95%CI | *P* |
| **Age** | 1.75 | 0.66-4.63 | 0.26 |  |  |  |  |
| **Gender** | 0.36 | 0.11-1.18 | 0.09 |  |  |  |  |
| **Lymphatic-vascular invasion** | 20.26 | 4.29-95.62 | <0.01 |  | 20.83 | 2.20 – 200.00 | <0.01 |
| **Grade** | 2.40 | 0.82-6.99 | 0.11 |  | 8.55 | 0.85-83.33 | 0.07 |
| **Multiple tumors** | 7.27 | 2.18 -24.22 | <0.01 |  | 10.00 | 0.94-111.11 | 0.06 |
| ***NRN1* methylation** | 49.00 | 11.92-201.49 | <0.01 |  | 111.11 | 7.58-1000.00 | <0.01 |
| **Maximum tumor size** | 1.59 | 0.61-4.19 | 0.35 |  | 4.44 | 0.31-62.50 | 0.27 |
| **Histologic variants** | 2.67 | 0.76-9.35 | 0.13 |  | 2.78 | 0.18=41.67 | 0.46 |
| **Risk-stratification model** | 1384.11 | 77.87-24600.63 | <0.01 |  |  |  |  |

OR: odds ratio; CI: confidence interval.

**Materials and Methods**

*Patient cohorts and study design*

We analyzed 124 patient samples from 2 independent cohorts: a training cohort of 78 patients were enrolled from five centers (Xiangya Hospital, the Second Xiangya Hospital, the Third Xiangya Hospital, Hunan Provincial People’s Hospital, Hunan Cancer Hospital), and a validation cohort of 46 patients from Haikou People’s Hospital. The study was approved by the institutional review boards of all participating institutions (ChiCTR2100043328), and written informed consent was obtained from all participants. Indications for re-TURBT were: incomplete initial TURBT, no muscle tissue in the first resection specimen, high-risk tumors, T1 tumor, G3/high-grade tumor (except carcinoma in situ). Exclusion criteria were synchronous advanced MIBC. The clinical T stage of the initial tumor was determined by CT or MRI. Re-TURBT was performed in all patients between 10 February 2021 and 10 April 2022. All specimens were evaluated by pathologists, according to the Seventh Edition of the American Joint Committee on Cancer TNM grading system, and minor histologic variants (<10% overall) are acceptable. The study was conducted in accordance with the Declaration of Helsinki. All enrolled patients underwent standard TURBT procedures and immediate single intravesical chemotherapy followed initial TURBT (within 24 hours). Re-TURBT was performed within 4-6 weeks of the initial TURBT. All TURBT procedures were performed by experienced urologists. Patients with high-risk tumors underwent intravesical Bacille Calmette-Guerin instillation was preformed 2 weeks after re-TURBT. For patients with intermediate-risk tumors, intravesical chemotherapy or intravesical Bacille Calmette-Guerin immunotherapy is recommended. The patients should first be given induction instillation for 6 weeks (once a week), followed by intensive instillation for 1.5 months (biweekly), followed by maintenance instillation for 10 months (once a month).

*Sample collection and DNA extraction*

Sample collection and DNA extraction methods were described in our previous study. A 50 mL morning urine midstream specimen of urine was collected from each participant before re-TURBT, stored at 4℃ and processed within 12 hours of collection. DNA was extracted using the GenMag Circulating Nucleic Acid Kit according to the manufacturer’s protocol and quantified using Qubit3.0.

*Methylation analysis*

The DNA methylation status was determined by methylation-specific polymerase chain reaction (MS-PCR). MS-PCR was performed according to standard protocols. ACTB was set as the internal reference. The relative methylation quantity of the internal reference gene and CpG markers were measured separately by FAM and VIC signals and represented as Ct values.

*Follow-up*

Cystoscopy was performed every 3 months for the first year, every 6 months for the second year, and once every year thereafter. CT or MRI was performed every 6 months. Comprehensive examinations were performed when symptoms worsened. The NRN1 methylation status and clinical data were assessed in a blinded manner. Independent statisticians were the first persons to unblind the data at the end of follow-up.

*Statistical analysis and logistic regression model*

All statistical analyses were performed using SPSS, v.24, GraphPad Prism v.8 and R 3.5.0. The characteristics of participants were compared by the t-test for continuous variables, the Mann–Whitney U test for non-normally distributed variables and the Chi-squared test for categorical variables. Logistic regression analysis was performed to evaluate the association between pathological features and variables. The AUC was used to evaluate the performance of models both in the training sets. P<0.05 was considered significant.
